# GLOBAL TRENDS IN FALL-RELATED INJURIES: A THREE-DECADE ANALYSIS

**DOI:** 10.1093/geroni/igae098.2715

**Published:** 2024-12-31

**Authors:** Ko Harada, Toshihiro Koyama, Yuji Yamada

**Affiliations:** Icahn School of Medicine at Mount Sinai, New York, New York, United States; Okayama University, Okayama, Okayama, Japan; Icahn School of Medicine at Mount Sinai, New York, New York, United States

## Abstract

**Background:**

Falls pose a significant public health challenge in our aging world, resulting in 684,000 deaths annually. Understanding the current situation is essential for developing data-driven policies and providing appropriate healthcare services. However, studies analyzing global trends in the disease burden of falls are scarce.

**Methods:**

This study utilizes the Global Burden of Disease database to explore the disease burden of falls between 1990 and 2019, focusing on mortality rates, incidence, and disability-adjusted life years (DALYs). Falls were defined as ICD-9 codes E880-E886, E888, and ICD-10 codes W00-W19.9, and the data was analyzed by regions based on the World Bank regional classifications.

**Results:**

Over the past 30 years, global deaths from falls per 100,000 population have increased by 29%, from 7.5 to 9.7. However, the global age-standardized mortality rate and age-standardized incidence rate from falls decreased by 8.4% and 2.8%, respectively. Trends in age-standardized mortality rate varied by region, with North America showing the most significant increase of 53%. Although age-standardized DALYs decreased by 14% worldwide, there was a 6.3% increase in North America. Conclusions: Our study showed that global fall-related mortality has increased worldwide over the past three decades, and the disease burden from falls has increased, particularly in North America, even after age adjustment. These trends can be attributed to the increase in multimorbidity, the prevalence of polypharmacy among older adults, and improved reporting quality. Developing and implementing targeted prevention strategies and policies is imperative to reduce the social impact of unintentional falls.

